# Deep transcriptome sequencing provides new insights into the structural and functional organization of the wheat genome

**DOI:** 10.1186/s13059-015-0601-9

**Published:** 2015-02-10

**Authors:** Lise Pingault, Frédéric Choulet, Adriana Alberti, Natasha Glover, Patrick Wincker, Catherine Feuillet, Etienne Paux

**Affiliations:** INRA UMR1095 Genetics, Diversity and Ecophysiology of Cereals, 5 chemin de Beaulieu, 63039 Clermont-Ferrand, France; University Blaise Pascal UMR1095 Genetics, Diversity and Ecophysiology of Cereals, 5 chemin de Beaulieu, 63039 Clermont-Ferrand, France; CEA/DSV/IG/Genoscope, 2 rue Gaston Cremieux, 91000 Evry, France; CNRS UMR 8030, 2 rue Gaston Crémieux, 91000 Evry, France; Université d’Evry, CP5706 Evry, France; Current address: Bayer CropScience, Technologiepark 38, Zwijnaarde, 9052 Gent Belgium; Current address: Bayer CropScience, 3500 Paramount Parkway, Morrisville, NC 27560 USA

## Abstract

**Background:**

Because of its size, allohexaploid nature, and high repeat content, the bread wheat genome is a good model to study the impact of the genome structure on gene organization, function, and regulation. However, because of the lack of a reference genome sequence, such studies have long been hampered and our knowledge of the wheat gene space is still limited. The access to the reference sequence of the wheat chromosome 3B provided us with an opportunity to study the wheat transcriptome and its relationships to genome and gene structure at a level that has never been reached before.

**Results:**

By combining this sequence with RNA-seq data, we construct a fine transcriptome map of the chromosome 3B. More than 8,800 transcription sites are identified, that are distributed throughout the entire chromosome. Expression level, expression breadth, alternative splicing as well as several structural features of genes, including transcript length, number of exons, and cumulative intron length are investigated. Our analysis reveals a non-monotonic relationship between gene expression and structure and leads to the hypothesis that gene structure is determined by its function, whereas gene expression is subject to energetic cost. Moreover, we observe a recombination-based partitioning at the gene structure and function level.

**Conclusions:**

Our analysis provides new insights into the relationships between gene and genome structure and function. It reveals mechanisms conserved with other plant species as well as superimposed evolutionary forces that shaped the wheat gene space, likely participating in wheat adaptation.

**Electronic supplementary material:**

The online version of this article (doi:10.1186/s13059-015-0601-9) contains supplementary material, which is available to authorized users.

## Background

In angiosperms, genome size is extremely variable, ranging from 63 Mb in *Genlisea margaretae* to 148,900 Mb in *Paris japonica*, that is, a 2,400-fold difference [1]. By contrast, the gene content seems relatively constant, with an average number of 30,000 and a two- to three-fold difference per diploid genome [2,3]. As a consequence, the gene space organization differs strikingly from one genome to another. For example, plants with small genomes such as *Arabidopsis thaliana* (125 Mb) and *Brachypodium distachyon* (272 Mb) exhibit an even distribution of their genes along their chromosomes [4] whereas for plants with intermediate size genomes such as *Populus trichocarpa* (485 Mb) and *Vitis vinifera* (487 Mb), alternation between high gene density regions and low gene density regions is observed [5,6]. This tendency is even stronger in plants with large genomes such as *Glycine max* (1,115 Mb) and *Zea mays* (2,300 Mb) in which a positive gradient of gene density from the centromere to the telomeres has been observed [7,8]. Beside the overall organization of genes, several studies revealed a non-random distribution of genes along chromosomes, resulting in clusters of genes sharing the same expression profile, the same function or involved in the same metabolic pathway [9-15]. In addition, relationships between gene structure and expression were reported in various organisms [16-18]. Altogether, these studies suggest a high degree of organization in gene space and interplay between genome and gene structure, function, and regulation.

With 220 million hectares, bread wheat (*Triticum aestivum* L.) is the most widely grown and consumed crop worldwide providing staple food for 30% of the world population. Beside its socioeconomic importance, bread wheat is also a good model for studying complex genome species. Indeed, with its large 17-Gb, allohexaploid (6x = 2*n* = 42, AABBDD) and highly repetitive (>80% transposable elements) genome, wheat is one of the most complex crop species. Other species share some of these features, but none of them, at least among cultivated species, combine the three. For example, the loblolly pine genome is the largest genome sequenced so far (22 Gb) but it is diploid [19]. Cotton is a polyploid species but has a smaller genome (2.5 Gb) [20] and so far only wild diploid relatives were sequenced [21,22]. The maize and sorghum genomes are highly repetitive but are diploid and smaller in size [7,8].

The wheat gene space organization and expression have been extensively investigated in the past decades. Many expression analyses have been conducted using either microarrays or RNA-seq but most of them were aiming at deciphering specific processes, such as grain development or response to stresses (for examples, see [23-27]). Other studies aimed at studying the gene space organization and reported on the existence of a gene gradient along the centromere-telomere axis as well as an organization of genes in small gene islands and co-expression/co-function clusters (for examples, see [28-30] and references therein). However, very few of these studies really investigated the relationships between genome and gene structure and function, mainly because of the lack of a reference genome sequence. The access to physical maps of wheat chromosomes provided the first opportunities to study gene regulation with respect to their physical position [29] although there were still limited to efficiently address this question. Recently, several initiatives aimed at generating draft genome sequences of hexaploid wheat or its diploid progenitors [21,22,31,32]. While they provided a quite comprehensive catalogue of wheat genes as well as novel data on gene evolution and expression, the highly fragmented nature of the sequence assemblies limits our ability to decipher the relationships between genome organization and gene regulation.

Recently, we have produced a 774-Mb reference sequence of the hexaploid wheat chromosome 3B [33]. Sequence annotation predicted 7,264 genes that were distributed along the chromosome with a gradient of density from centromere to telomeres. The distribution of structural and functional features along the chromosome revealed partitioning correlated with meiotic recombination. Three main regions were identified: two distal regions of 68 Mb (region R1; from 1 to 68 Mb) and 59 Mb (region R3; from 715 to 774 Mb) on the short and long arms, respectively, and a large proximal region of 648 Mb (region R2; from 68 to 715 Mb) spanning the centromere. In addition, we delineated a 122-Mb central region (from 265 to 387 Mb), enriched in centromere-specific transposable elements, as the centromeric-pericentromeric region of chromosome 3B.

Here, we report a detailed analysis of the chromosome 3B transcriptional landscape. By combining deep transcriptome sequencing data covering the whole plant development with the reference sequence of the chromosome, we identified transcriptionally active regions distributed throughout the entire chromosome. Relationships between genome and gene structure and function revealed different mechanisms governing the gene space organization, regulation, and evolution.

## Results and discussion

### Chromosome 3B contains more than 8,800 transcriptionally active regions

To study the expression profiles of hexaploid wheat chromosome 3B genes during the life cycle of a wheat plant and establish a transcriptome atlas for this chromosome, deep transcriptome sequencing was conducted in duplicates in 15 wheat samples corresponding to five different organs (leaf, shoot, root, spike, and grain) at three developmental stages each [28]. Strand-non-specific and strand-specific libraries were used to produce 2.52 billion paired-end reads (232 Gb) and 615.3 single-end reads (62 Gb), respectively. The reads were then mapped to the chromosome 3B reference sequence [33], without allowing for any mismatches in order to discriminate chromosome 3B expressed genes from homoeologous and paralogous copies. Eventually, 3.66% of reads mapped onto chromosome 3B of which 98% were mapped uniquely. Ninety-five percent of the reads matched sequences annotated as genic regions whereas the remaining 5% mapped to regions where no protein-coding gene was predicted by the annotation [33].

Within the 774.4 Mb comprising the pseudomolecule of chromosome 3B, 8,877 transcriptionally active regions (TARs) were identified, corresponding to an average density of one TAR every 87 kb (Table 1). Among these, 5,185 corresponded to predicted gene models, including pseudogenes and gene fragments [33]. This represents 71.4% of the 7,264 predicted gene models. The genes contained on average 4.6 exons, ranging from one to 53, which is similar to what was found in *B. distachyon* (5.2), rice (3.8), maize (4.1), sorghum (4.3), and *Triticum urartu* (4.7) [8,21,34-36]. The percentage of expressed genes is slightly lower than the ones reported in other plant species. Indeed, a microarray analysis of the rice transcriptome performed in seedling shoots, tillering-stage shoots and roots, heading, filling-stage panicles, and suspension-cultured cells detected expression for 86% of the 41,754 known and predicted gene models present on the microarray [37]. More recently, Lu *et al.* [38] conducted an RNA-seq analysis on seeds from three rice cultivated subspecies and found that 83.1% of the 46,472 annotated gene models were expressed. Similarly, in maize, microarray-based transcript profiling in 60 distinct tissues representing 11 major organ systems revealed that 91.4% of the genes were expressed in at least one tissue [39]. More recently, Sekhon and colleagues [40] performed RNA-seq experiments on a subset of 18 selected tissues representing five organs and showed that 74.7% of the 39,429 genes from the filtered gene set were transcribed. In soybean RNA-seq analysis revealed that 80.4% of 69,145 putative genes are expressed in a least one of the 14 tissues analyzed [41]. The lower percentage of genes expressed in wheat might suggest a small impact of polyploidization on gene silencing. This is consistent with previous studies conducted in newly synthesized polyploid wheat and rapeseed where 7.7% and 4.1% of the sequences showed alteration in gene expression [42,43]. To estimate the exact extent of gene silencing in hexaploid wheat, a comparison with diploid and tetraploid progenitors would be required. However, when considering only genes likely to be functional (hereafter referred to as ‘full genes’), the percentage of expressed genes rose to 77.5% (4,125/5,236), which is similar to the percentages found in maize and soybean using a similar number of conditions [40,41]. Beside full genes, 54.7% (1,060/1,938) regions annotated as pseudogenes or gene fragments in the pseudomolecule were found to be expressed in at least one condition. In other species such as *A. thaliana* and rice, EST analyses revealed expression for 2% to 5% and 2% to 3% pseudogenes, respectively [44]. Another study conducted on 1,439 rice pseudogenes using Massively Parallel Signature Sequencing tags suggested that up to 12% are expressed in at least one of the 22 samples studied [45]. These proportions strongly differ from our results. One cannot exclude that the percentage of pseudogenes expressed on chromosome 3B could be overestimated as a result of the RNA-seq technology that cannot completely discriminate pseudogene expression from close functional copies that might be present elsewhere in the genome. In an attempt to assess this overestimation, we searched the recently released draft assembly of the wheat genome [31] for additional copies of pseudogenes in the genome. Overall, 511 out of 1,060 (48.2%) had at least one other copy, whereas 51.8% were found to be present in one single copy located on chromosome 3B. Assuming that for the 48.2% ‘multicopy’ pseudogenes, transcripts were not produced by the 3B loci, our results suggest that 28% of the chromosome 3B pseudogenes are still expressed, which is much higher than what has been observed in other organisms so far. Transposable elements (TEs) have been shown to be able to generate sense or antisense transcripts of adjacent genes [46]. Given the high proportion (>85%) of the wheat genome covered by TEs, one can hypothesize that some TEs provide a promoter for transcription of adjacent pseudogenes. In addition, while they have long been considered as non-functional units, several studies suggest that pseudogenes might play a role in regulation through antisense regulation of their parental gene, competition for miRNA, generation of small-interfering RNA, or production of short proteins or peptides [47,48]. The high percentage of expressed pseudogenes found in wheat compared to other species might therefore be due to their role in the regulation of homoeologous or paralogous gene expression.

**Table 1:** **General features of the chromosome 3B pseudomolecule transcriptionally active region**

**Transcriptionally active regions**		**Predicted** ^**a**^	**Expressed**
Protein-coding genes			
	Total	7,264	5,185
	Full genes	5,326	4,125
	Pseudogenes and fragments	1,938	1,060
Novel transcribed regions			
	Total	-	3,692
	Putative lincRNAs	-	1,922
*cis-*NATs			635

^a^According to [33].

For 28.6% of the predicted gene models (2,079), we failed to detect any expression. This result probably reflects the fact that these genes might be expressed in specific conditions that have not been studied in the present work. Indeed, a Gene Ontology term analysis of these non-expressed genes revealed enrichment in biological processes such as ‘gametophyte development’, ‘response to temperature stimulus’, or ‘response to water; (Additional file 1: Table S1). In addition, 57.5% of the non-expressed genes are non-syntenic with *B. distachyon*, rice, and sorghum, suggesting that some of these genes might have been duplicated and translocated without their regulatory sequences, leading to their transcriptional inactivity. Finally, it is worth noting that the proportion of non-expressed pseudogenes is twice as high as the proportion of non-expressed functional genes (45.3% *vs.* 22.6%). As a result, the distribution pattern of non-expressed genes along the chromosome was found to be highly correlated with that of pseudogenes (r_*S*_ = 0.81, *P* <2.2e10^−16^), and even more with that of single copy pseudogenes (r_S_ = 0.86, *P* <2.2e10^−16^).

In addition to the predicted gene models, expression was detected for 3,692 loci in unannotated regions. These so-called novel transcribed regions (NTRs) represented on average 22% of all TARs. Twenty-eight percent (1,033/3,692) of these NTR-translated sequences shared weak similarity with plant proteins, mainly TE-encoded proteins or hypothetical proteins and might therefore be protein-coding genes (or pseudogenes). Out of the 2,659 with no similarity with plant proteins, 596 were longer than 200 nt and did not carry ORFs longer than 300 AA. These NTRs might therefore correspond to long intergenic non-coding RNAs (lincRNAs) as defined by Liu and colleagues [49]. Based on this number, one could speculate that roughly 10,000 lincRNAs should be expressed in the whole wheat genome, or 3,300 per diploid genome. This number is comparable to that of expressed lincRNAs reported *A. thaliana* [49] and poplar [50] (2,708 and 2,542, respectively). Out of these 596 putative lincRNAs, 91.1% and 93% were found in the *Triticum urartu* and *Aegilops tauschii* genomes, respectively. The percentage decreases to 69.3% when looking at the barley genome. An even more drastic drop was observed when moving out of the Triticeae tribe, with only 14.8%, 7%, and 6.2% of the putative lincRNAs conserved in the *B. distachyon*, rice, and sorghum genomes, respectively. These findings suggest that most of these putative lincRNAs are functional elements that have been acquired by the wheat and more largely the Triticeae genomes in the time course of their evolution.

Beside lincRNAs that are located in intergenic regions and therefore do not overlap with protein-coding genes, *cis-*natural antisense transcripts (NATs) are another form of long non-coding RNAs [51-53]. To estimate the extent of *cis*-NATs in wheat, oriented RNA-seq libraries from the five organs were constructed and reads were mapped on chromosome 3B without allowing mismatches. Out of the 5,185 expressed genes, 635 (12.2%) were found to be transcribed on the reverse strand as well, therefore producing a *cis*-NAT. It is worth noting that *cis*-NATs originate preferentially from syntenic genes (72.4%) and the vast majority (84.9%) concerned full genes. A previous study conducted in wheat using microarray identified 110 NATs at the whole genome level [54]. Conversely, Serial Analysis of Gene Expression showed that up to 25.7% of wheat was represented by reverse tags [55]. Such widespread occurrence of antisense transcription has already been reported in other plant species such as *A. thaliana*, rice or maize where 2.8% to 9.7% of genes produce antisense transcripts [53,56,57]. *Cis*-NATs can regulate gene expression at the transcriptional or post-transcriptional level through various mechanisms [51,58]. In a polyploid species, one can hypothesize that they play a role in the regulation of homoeologous copies.

### Transcription sites are distributed throughout the entire chromosome 3B

**Figure 1:**
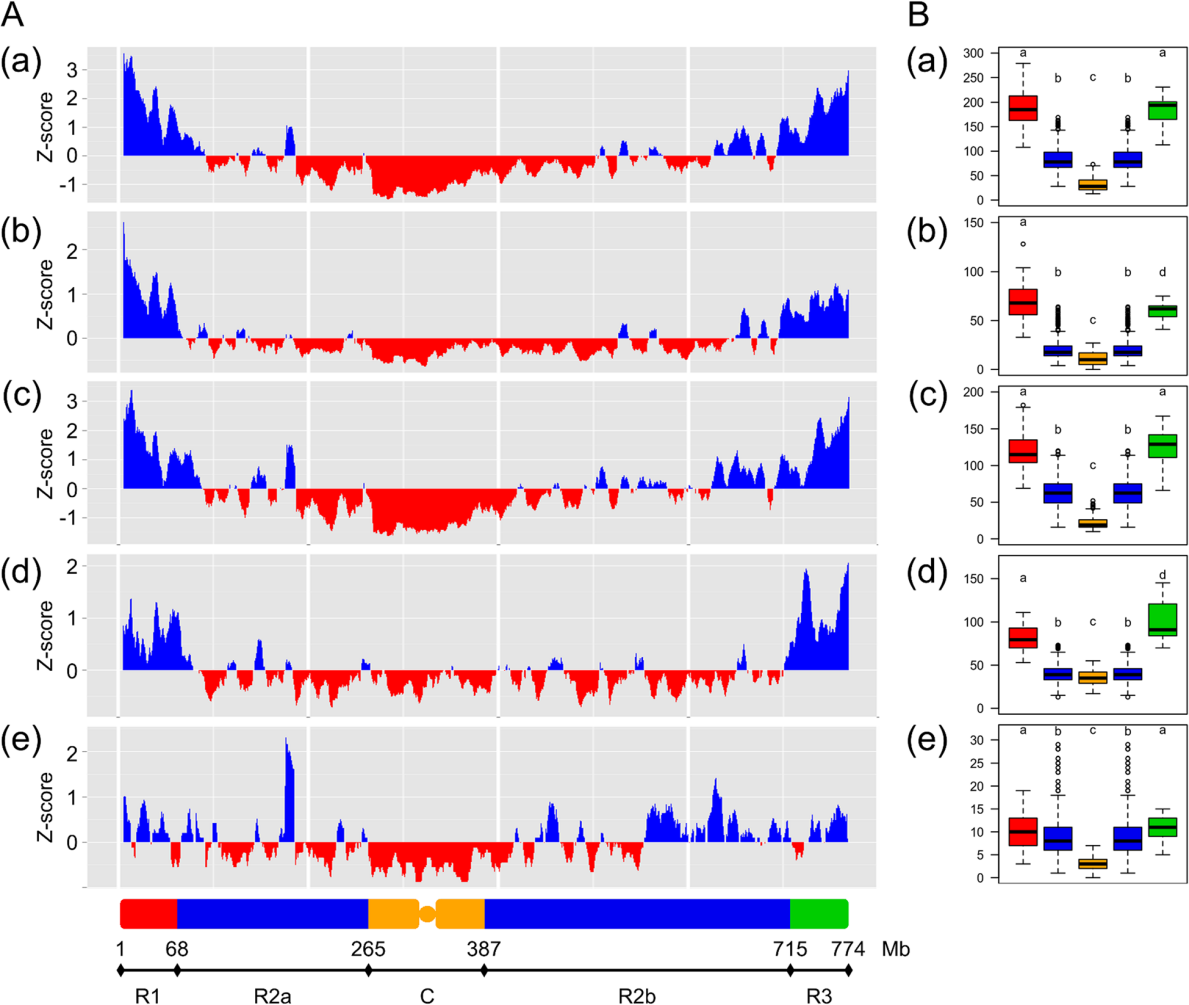
**Distribution of the functional regions on the chromosome 3B. (A)** Z-score of the feature density of in a 10-Mb sliding window (step 1 Mb) along chromosome 3B. Positive values are in blue, negative values in red. Features include (a) predicted genes, (b) non-expressed predicted genes, (c) expressed predicted genes, (d) NTRs, (e) *cis*-NATs. The five main regions of the chromosome 3B are depicted at the bottom of the graph: R1 in red; R2a and R2b in blue; C in orange; R3 in green. The borders of these regions are indicated in Mb. **(B)** Boxplots of the feature density in the five main regions of the chromosome 3B (R1 and R3 in blue; R2a and R2b in yellow; C in red). Features include (a) predicted genes, (b) non-expressed predicted genes, (c) expressed predicted genes, (d) NTRs, (e) *cis*-NATs.

The distribution of predicted protein-coding genes, non-expressed genes, expressed genes, NTRs, and cis-NATs density was analyzed along chromosome 3B (Figure 1A). Recently, Choulet *et al.* [33] reported on the structural and functional partitioning of chromosome 3B based on recombination pattern. While the R1 and R3 regions tended to be quite homogeneous, the R2 region appeared to be highly heterogeneous in terms of transposable element and gene content, expression breath as well as linkage disequilibrium. This is especially true in the so-called centromeric-pericentrometic region. Thus to refine our analysis of chromosome 3B, we divided the chromosome in five regions: R1 (1 to 68 Mb), R2a (68 to 265 Mb), C (265 to 387 Mb), R2b (387 to 715 Mb), and R3 (715 to 774 Mb) (Figure 1B). The densities of predicted protein-coding genes, non-expressed genes, expressed genes, NTRs, and *cis*-NATs were then computed in each of these regions. The density of expressed genes was highly correlated with the distance to the centromere (r_*s*_ = 0.77, *P* <2.2e-16) and was found to follow that of predicted protein-coding genes (χ^2^ test = 415.84, df = 762). The overall average density was 6.5 ± 3.3 genes/Mb, ranging from 1.0 in the centromeric region up to 18.2 at the most telomeric end of the short arm. With an average density of 4.8 ± 4.1 per Mb, NTRs were slightly less abundant than expressed protein-coding genes but followed the overall gene distribution. However, their proportion was found to be much higher in the pericentromeric C region. Since this region corresponds to the part of the chromosome where the TE density is the highest, this suggests that some of these NTRs might actually be transcribed from adjacent TE promoters. Whether these RNAs are ‘transcriptional’ noise or have a biological function remains to be investigated. The distribution of *cis*-NATs is slightly more even along the chromosome, suggesting that proximal genes are more prone to antisense transcription than distal ones. Once again, this might be due to the high abundance of TEs in these regions that would provide promoters for the transcription of adjacent genes.

Taken together, these results clearly demonstrate that transcription occurs all along chromosome 3B and is not restricted to distal regions. This is in complete agreement with our previous analyses using microarray hybridizations of BAC pools and mRNA samples [29] and with observations from Abranches *et al.* [59] who demonstrated that active transcription sites are distributed throughout the wheat genome and do not show any preferential localization in the nuclei. More recently, Baker *et al.* [60] provided evidences that genes located in the low recombining pericentromeric regions were expressed at a level that was similar to that of genes in high recombining distal regions in barley. Thus, while gene density follows an increasing gradient along the centromere-telomere axis that correlates with recombination, this distribution does not seem to relate to the overall transcription capacities of wheat genes.

### Expression level, expression breadth, and alternative splicing are correlated

**Figure 2:**
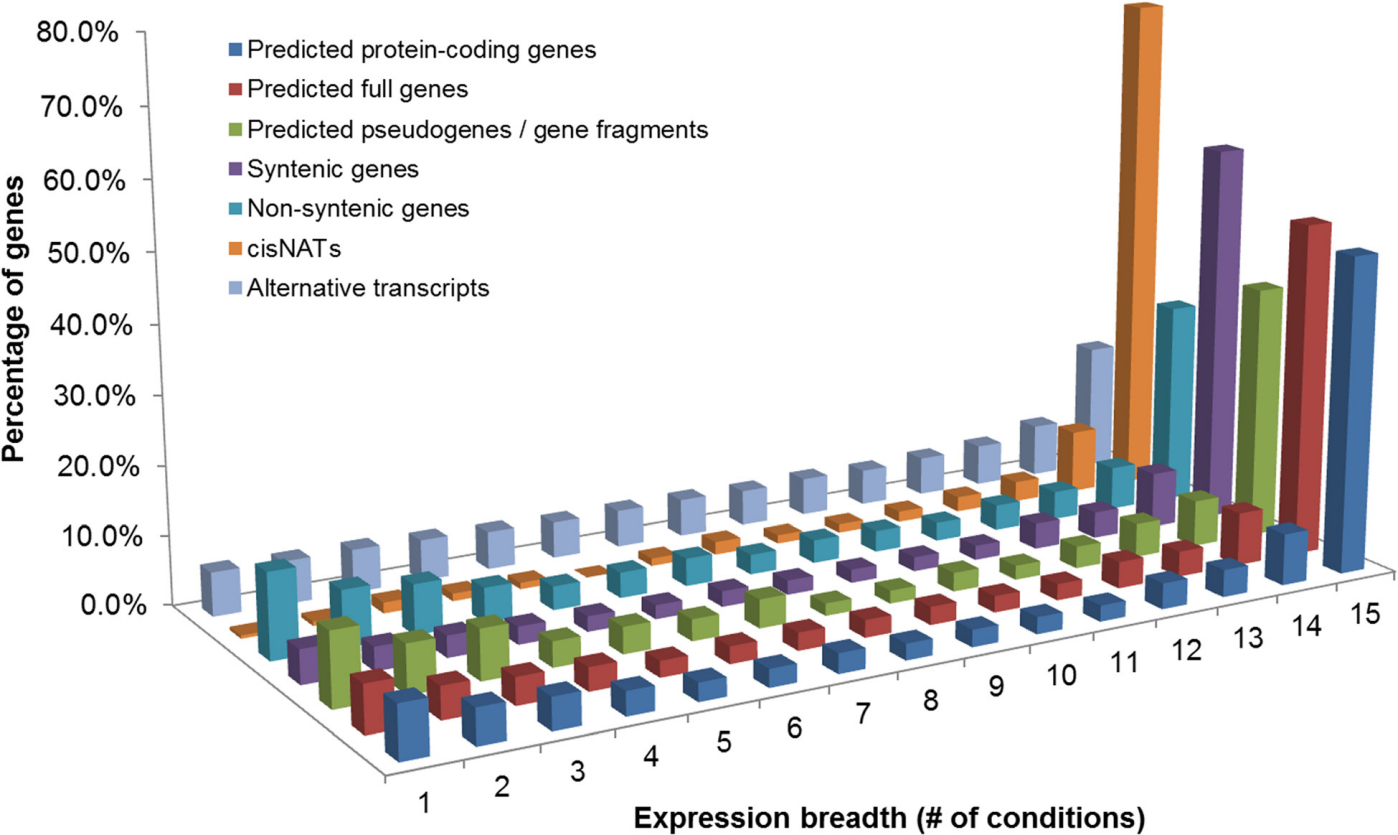
**Distribution of the percentage of transcriptionally active regions expressed in the different number of experimental conditions.** Regions were classified according to their expression breadth, that is, the number of conditions in which they were expressed, from 1 to 15. Dark blue: predicted protein-coding genes; red: predicted full genes; green: predicted pseudogenes/gene fragments; purple: syntenic genes; cyan: non-syntenic genes; orange: *cis*-NATs; light blue: alternative transcripts.

The number of expressed genes was found to be comparable across the 15 conditions, with on average 3,734 ± 228 genes expressed per condition. A similar trend was observed in other species such as maize [39], soybean [41], or peach [61]. The average expression breadth (that is, the number of conditions in which a gene is expressed) for the 5,185 expressed gene models was 10.8, with 46.2% (2,396) of the genes expressed in all conditions and 7.6% (396) exhibiting a condition-specific expression profile. At the organ level, the number of organ-specific genes ranged from 77 in leaf to 243 in spike. These proportions of condition-specific genes are not similar for all types of genes (Figure 2). For example, pseudogenes and gene fragments were found to be more specific than full genes with only 36.9% of them being expressed in 15 conditions and 10.7% in one single condition (*vs.* 48.6% and 6.9% for full genes, respectively). A similar trend was observed when comparing syntenic and non-syntenic genes that were identified by comparative analysis along the 3B sequence [33]. Indeed, 29.6% of the non-syntenic genes were expressed in 15 conditions and 12.5% in one single condition, whereas 55.5% and 4.9% of syntenic genes were found to be expressed in 15 and one condition, respectively. By contrast, 73.7% of genes showing anti-sense transcription were expressed in 15 conditions while only very few of them (0.5%) were specific to one single condition. This reinforces the idea that *cis*-NATs serve as post-transcriptional regulators of gene expression [62-64].

Expression breadth was found to be correlated with expression level. This correlation is not unexpected since genes that are widely expressed such as house-keeping genes tend to show a higher expression level than condition-specific genes [17,65,66]. However, to some extent, one cannot exclude that this relationship between expression level and expression breadth reflects the fact that expression is not detected in some conditions and that some condition-specific genes might just be low expression genes.

Our analysis revealed 30,232 transcripts originating from the 5,185 chromosome 3B expressed genes. Thirty-nine percent of the genes were transcribed in one single mRNA in our conditions whereas splicing variants were detected for 61.4%, with an average of 5.8 alternative transcripts per gene. When considering multiexonic genes only, the percentage of alternatively spliced genes raised to 75.4%. While alternative splicing (AS) is a general phenomenon in plants, the overall AS level differs strikingly between species. Indeed, previous studies reported that 61% and 48% of *A. thaliana* and rice genes undergo AS, respectively [38,67], whereas only 6.3% and 15.9% of expressed genes are under the potential influence of AS in *B. distachyon* and soybean, respectively [68,69]. In barley, 55% of high confidence genes and 73% of intron-containing high confidence genes have evidence of AS [70]. This high similarity between barley and wheat, as well as differences with that of rice and *B. distachyon* suggests that the AS level might have evolved differently in grasses. Conversely, considering that the level of AS observed in wheat was similar to that of *A. thaliana*, it is very unlikely that these differences between species are linked to genome size and complexity. However, one cannot exclude that differences originate from experimental design. In *A. thaliana*, the predicted AS level increased from 1.2% to 61% between 2003 and 2012, mainly as a result of the advent of high-throughput technologies [71]. In addition, alternative transcripts have been hypothesized to be tissue- or condition-specific [72]. As our results are based on the study of plants grown in normal conditions we cannot exclude that the percentage of AS genes is underestimated and might increase with the inclusion of other samples such as plants grown under stress conditions.

Beside the differences observed in the overall AS level between species, we found differences in the relative abundance of the main types of AS, namely exon skipping (ES), alternative splice sites (A3SS and A5SS), intron retention (IR), and mutually exclusive exons (MXE) [73]. In wheat, IR was found to be the predominant type, with 35% of all events, followed by A3SS (27%), ES (21%), A5SS (16%), and MXE (0.9%). In *A. thaliana*, rice, and *B. distachyon*, IR accounts for more than 50% [68,74] whereas the predominant type was found to be ES in the peach genome, with 43% of all observed events [61]. Such differences strongly reinforce the idea that, despite the fact that AS is a common phenomenon shared by most if not all plant species, specificities have been acquired by the different plant species during the course of their evolution.

While 46.2% of the 5,185 genes were expressed in 15 conditions, only 18.6% of 30,232 transcripts appeared to be present in all conditions, which is very similar to what has been observed in barley [70]. In addition, 95% of the AS transcripts originating from the same gene exhibited different expression profiles, as revealed by a hierarchical clustering of the 30,232 transcripts (data not shown). As a consequence, the number of alternative transcripts was found to be positively correlated with the expression breadth. These findings strongly suggest that AS variants have complementary functions across organs or developmental stages.

### A non-monotonic relationship between gene expression and gene structure

A negative correlation was observed between the transcript size and the expression breadth, with shorter transcripts being expressed in more conditions. This is consistent with previous studies indicating that house-keeping genes which are expressed in more conditions are generally more compact than genes expressed in specific conditions [17,65,66,75]. Such findings could be explained by the ‘selection for economy’ model [17,76]. In this model transcription and translation are both time- and energy-consuming and, as a consequence, widely and highly expressed genes tend to be more compact to reduce the energetic cost [77,78].

**Figure 3:**
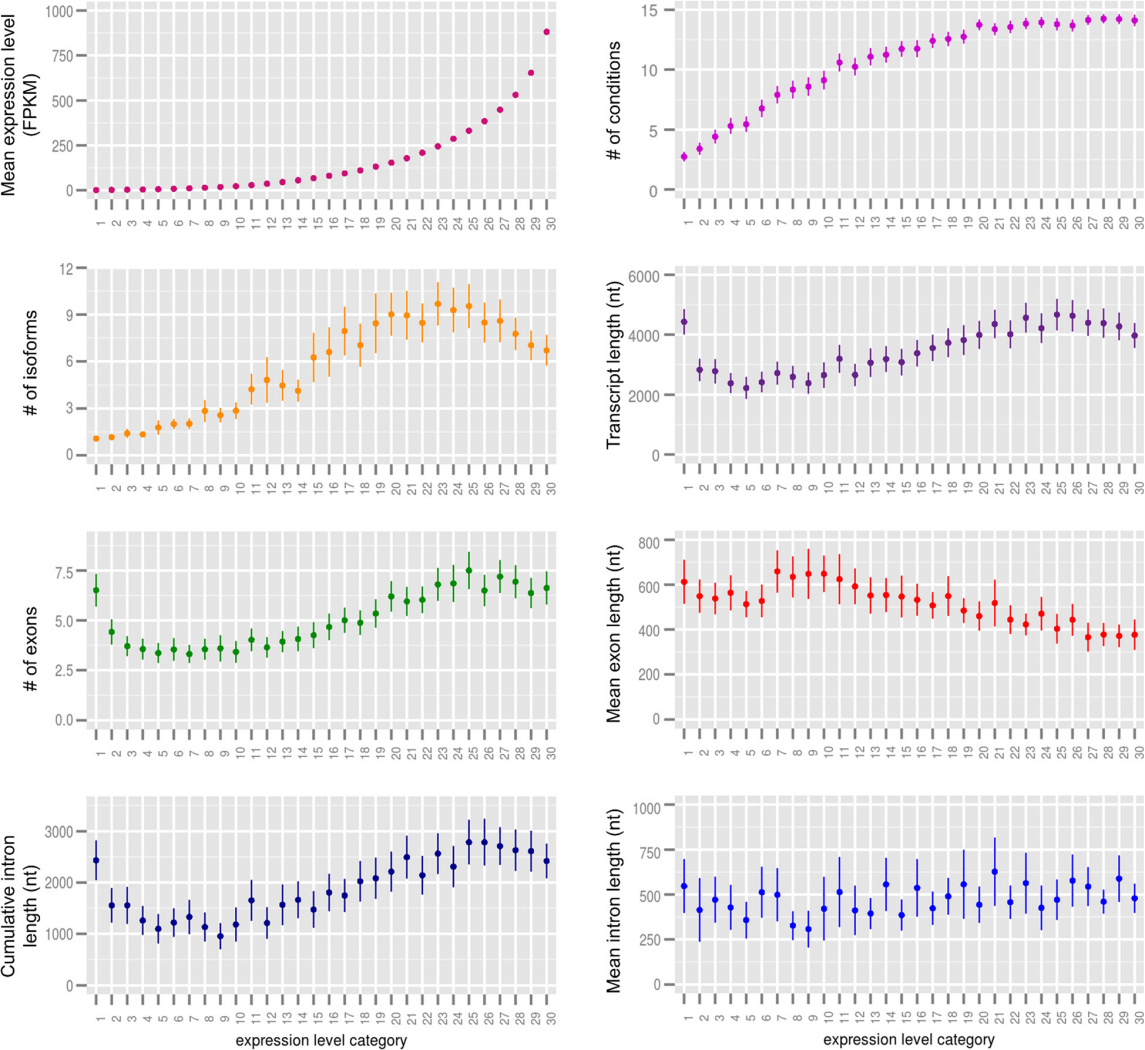
**Relationships between gene expression and gene structural and functional features.** Expression levels are binned into 30 categories. Each dot is the mean value for genes in the given expression category, and the error bar indicates the standard deviation of the mean.

We then investigated the correlation between expression level and gene structure, in terms of transcript size, number of exons, cumulative intron length, mean exon and intron length, and number of alternative transcripts. To this aim, genes were grouped in 30 classes of similar size based on their average expression level (that is, the average FPKM value in a number of conditions where the gene is expressed), as done by Carmel and Koonin [16]. Then, the average values of different variables in each of the 30 expression level classes were computed across the genes (Figure 3).

For the transcript size, the number of exons, the cumulative intron length, and the number of alternative transcripts, non-monotonic relationships were found with the expression level, resulting in an approximate bell-shaped dependence (Figure 3). For all features, the area of the inflexion point was comprised between classes 20 and 25 which is also the area where expression breadth reaches a plateau. Following criteria defined by Hansey *et al*. [79], genes in categories 1 to 4 correspond to low expression genes (mean expression level <5 FPKM), those in categories 5 to 21 to medium expression genes (5 ≤ mean expression level <200 FPKM) and those in categories 22 to 30, to high expression genes (mean expression level ≥200 FPKM). Interestingly the inflexion point also corresponded to the threshold between medium and high expression genes. For medium expression genes, the expression level was positively correlated with the structural features whereas for high expression genes, we found a negative correlation. For low expression genes, the observed relationship might be an artifact resulting from the detection threshold of low abundance transcripts. Indeed some of these genes might have been considered as expressed when actually they were not. Therefore, these four classes (1 to 4) might not be reliable as they might contain a mix of expressed and non-expressed genes leading to average structural feature values that are not representative of expressed genes. For the mean exon and intron length, no clear relationship was observed even though the mean exon length tends to decrease as the expression level increases. Such a non-monotonic relationship has already been observed in other organisms, including human, *Caenorhabditis elegans*, *Drosophila melanogaster*, *A. thaliana*, and soybean [16,18]. If the ‘selection for economy’ fits for highly expressed genes, it cannot apply to low to medium expression genes. The ‘genome design’ model has been proposed to explain this relationship [17,66,75,76]. It suggests that the structural features of a gene are mostly determined by its functional load. Highly and widely expressed genes would not require a fine regulation and therefore less regulatory sequences. By contrast, for low/medium expression, condition-specific genes, longer intragenic non-coding sequences would allow for a more complex regulation. Since the number of alternative transcripts follows the same distribution, one can hypothesize that the greater number of exons and the larger intronic sequences might allow for a greater transcriptional complexity leading to a greater specificity in gene expression. A detailed analysis of transcript size based on the 30,232 isoforms showed a negative correlation with expression level, regardless of the expression class (Additional file 2: Figure S1). This finding reinforces the hypothesis that gene structure would be determined by its function (‘genome design’ model) whereas the expression of the different transcripts from a given gene would be subject to the energetic cost (‘selection for economy’ model).

### Gene structural and functional features are partitioned on chromosome 3B

**Figure 4:**
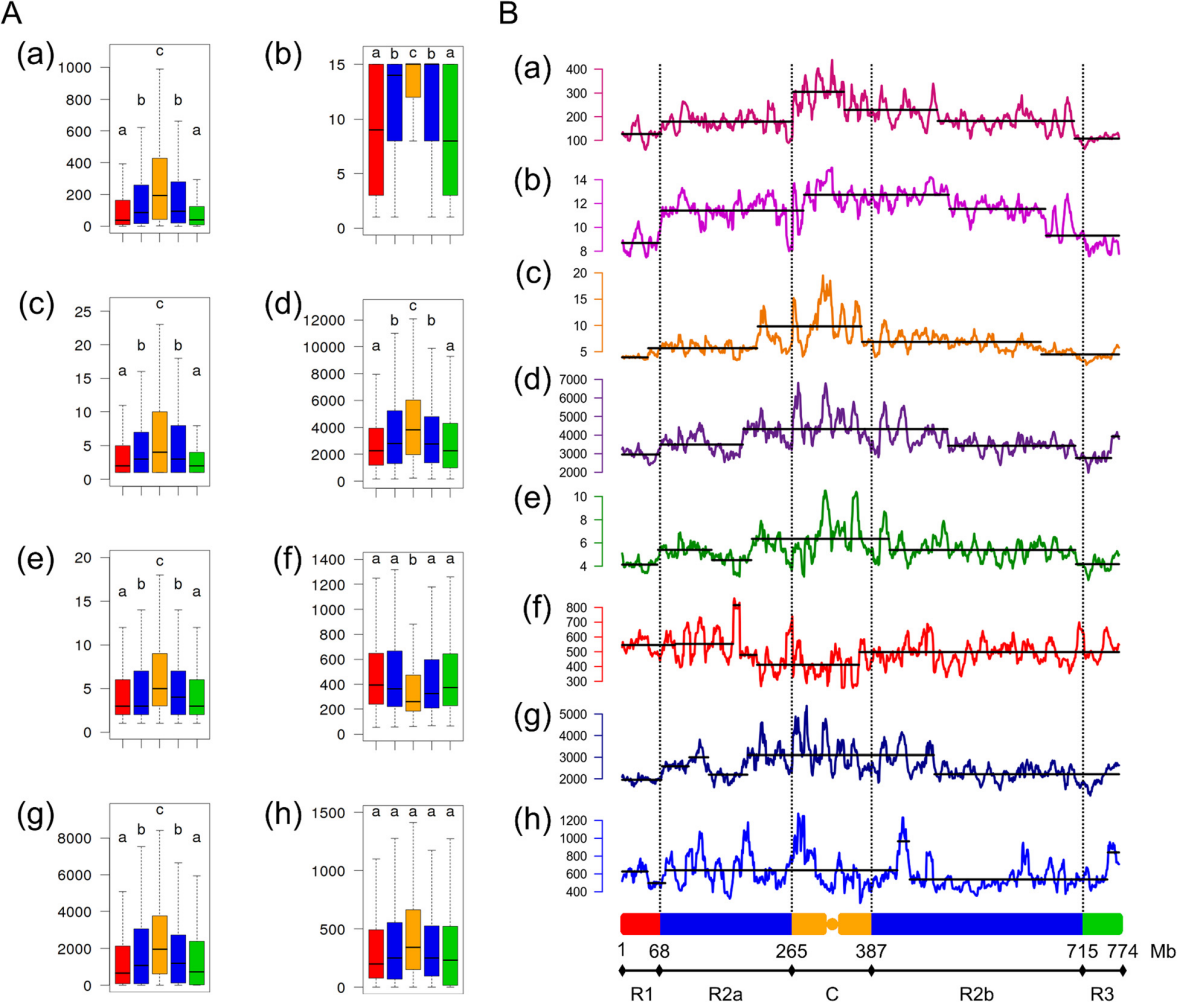
**Distribution and functional partitioning of wheat chromosome 3B. (A)** Boxplots of structural and functional gene features in the five main regions of the chromosome 3B (R1 in red; R2a and R2b in blue; C in orange; R3 in green). (a) Gene expression in FPKM; (b) expression breadth in number of conditions; (c) number of alternative transcripts per gene; (d) transcript length; (e) exon number; (f) mean exon length; (g) cumulative intron length; (h) mean intron length. **(B)** Distribution and segmentation analysis of (a) gene expression in FPKM, (b) expression breadth in number of conditions, (c) number of alternative transcripts per gene, (d) transcript length, (e) exon number, (f) mean exon length, (g) cumulative intron length, (h) mean intron length. Sliding window size: 10 Mb, step: 1 Mb. The five main regions of the chromosome 3B are depicted at the bottom of the graph: R1 in red; R2a and R2b in blue; C in orange; R3 in green.

To investigate their relationship with chromosome partitioning, the expression breadth, expression level, transcript size, number of exons, cumulative intron length, mean exon and intron length, and the number of alternative transcripts were computed in the five regions of chromosome 3B, namely R1, R2a, C, R2b, and R3 (Figure 4A). For all features but mean exon and intron length, the regions can be classified in three contrasting groups. The first one includes regions R1 and R3, the second one, R2a and R2b, and the third one, C. All of the features decrease along the centromere-telomere axis. Thus, on average, genes in distal regions are expressed at a lower level, more specifically and have fewer isoforms than those in the proximal regions. In addition, they are shorter, with fewer exons and shorter intronic sequences. Genes located in region C tend to have shorter exons, while for the mean intron length, no significant differences were observed between the five regions. A segmentation analysis of these properties suggests a partitioning of the chromosome rather than a regular gradient from centromere to telomeres (Figure 4B). Interestingly, the boundaries of the distal segments fit almost perfectly with the R1 and R3 regions defined by Choulet *et al.* [33] based on recombination. It is worth noting that the analysis of chromosomes 3A and 3D based on their draft assembly [31] also revealed a strong partitioning of expression breadth, suggesting that the features observed on chromosome 3B should be conserved at the whole wheat genome level (Additional file 1: Figure S2).

To see to what extent the non-monotonic relationship between expression level and gene structural features observed at the whole chromosome level is conserved at the region level, we applied the same analysis to regions R1/R3 and R2a/R2b. Region C was not included due to the limited number of genes present in this region. Interestingly, the chromosomal pattern remained the same in each region (Additional file 1: Figures S3 and S4). Even though the average expression level was lower in R1/R3 regions, the mean expression level value of the inflexion point was conserved in the two regions, around 200 FPKM, the approximate threshold between medium and high expression genes. This finding clearly shows that the ‘selection for economy’ and ‘genome design’ models apply all along the chromosome independently of other features and strongly suggests that the evolutionary forces that have led to the chromosome partitioning are distinct from the molecular mechanisms governing gene expression.

### Chromosome conformation may play a role in gene regulation

**Figure 5:**
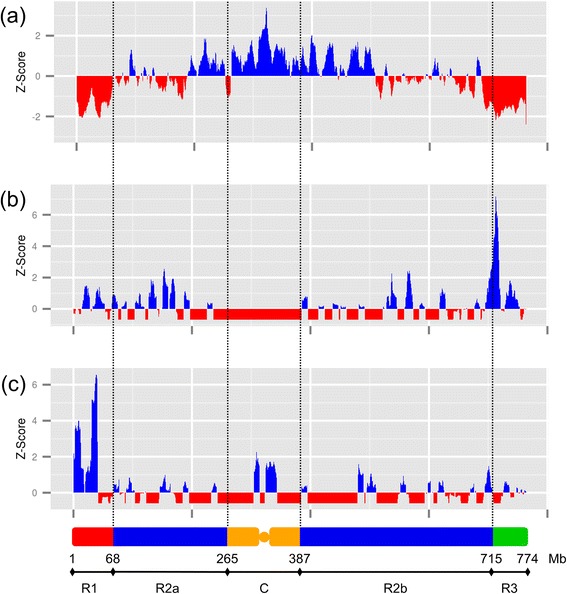
**Distribution of the percentage of genes from three different expression clusters.** Z-score of the percentage of expressed genes for a given cluster in a 10-Mb sliding window (step 1 Mb) along chromosome 3B. Positive values are in blue, negative values in red. **(a)** Constitutively expressed genes; **(b)** spike- and grain-specific genes; **(c)** genes preferentially expressed in leaf. The five main regions of the chromosome 3B are depicted at the bottom of the graph: R1 in red; R2a and R2b in blue; C in orange; R3 in green.

A hierarchical clustering of the 5,185 expressed protein-coding gene primary transcripts was performed based on their expression profiles in the 15 conditions. Genes were aggregated into 55 distinct clusters according to their expression profiles. Based on the median value of intergenic distances of 30 kb, Choulet *et al.* [33] estimated that 73% of genes were organized in small islands or ‘*insulae*’. Using the same criteria, 3,465 out of the 5,185 expressed genes (67%) were found to be organized in 1,199 *insulae*, comprising 2.9 genes on average. Out of these 3,465 genes, 1,218 (35.2%) belong to the same expression cluster as their direct neighbor, defining 718 co-expressed gene pairs. This proportion is higher than the previously reported value of 11% [29] most probably because of the higher resolution achieved with a reference sequence compared to a partial gene dataset. Such enrichment has already been reported in other organisms such as human, mouse, *A. thaliana*, rice, and fruit fly where the percentage of adjacent co-expressed genes is in the range of 2% to 20% [11,12,15,80,81]. However, these percentages are relatively low compared to that of wheat. One can hypothesize that the higher proportion of co-expressed genes found in wheat might result from the high rate of tandem duplication in this genome [33]. However, of the 718 co-expressed gene pairs, only 46 (6.4%) correspond to duplicated genes. This clearly shows that duplicate genes alone do not explain the observed levels of co-expression, as already reported in other organisms [9,13,15,82]. Several other mechanisms have been proposed to explain the co-expression of neighboring genes, including shared promoters and chromatin remodeling. In *A. thaliana*, Chen *et al.* [82] showed that co-expression was strongly enhanced for divergently transcribed genes within a 400-bp gene distance, probably as a result of shared promoters. For longer intergenic distances, co-expression is likely mediated by shared chromatin environments. On chromosome 3B, the average intergenic distance between co-expressed neighbor genes is 6.3 kb and only 133 out of the 718 gene pairs are transcribed divergently. This suggests that shared promoters are not the main mechanism controlling the co-expression of neighbor genes and that other mechanisms such as chromatin conformation might be involved. This hypothesis is reinforced by the significant differences observed for 23 out of the 55 expression clusters between the five regions (Additional file 1: Table S2). For example, the vast majority (63%) of the genes present in the region C belong to cluster I that correspond to genes expressed in all conditions whereas this cluster represents only 23% to 24% of region R1 and R3 genes (Figure 5). Region R1 is enriched in genes preferentially expressed in leaf compared to other regions. Region R3 displays a higher proportion of spike- and grain-specific genes. In addition, expression clusters oscillated along chromosome, forming chromosomal domains. These findings are consistent with the Gene Ontology term enrichment analysis that revealed that distal regions were enriched in genes involved in adaptive processes such as response to abiotic stimuli or stress [33].

Even though transcription sites are distributed throughout the entire chromosome when looking at the plant development at a whole, our results show that 3B is organized in chromosomal domains, suggesting that gene position influences the spatio-temporal regulation of their expression. Such domains have recently been reported for genes expressed in wheat endosperm [23]. While no overall subgenome dominance has been observed in wheat, abundant transcriptional dominance of subgenome segments as well as asymmetrical expression of neighboring genes were observed [23,31]. This strongly differs from other polyploid or paleopolyploid species such as cotton, *Brassica rapa* or maize in which a clear subgenome dominance was observed [83-85]. This specific pattern of the wheat genome suggests that, in this species, polyploidization might have impacted gene expression through the formation of dominant chromosome domains rather than overall subgenome dominance. In addition, it has been shown that the spatial organization of genomes in the interphase cell nucleus is tissue-specific [86]. This positioning of chromosomes is non-random and is likely to play a role in gene regulation [87,88]. In wheat, the interphase chromosomes are not fully decondensed but adopt a regular Rabl configuration, a highly polarized pattern with the two chromosome arms lying next to each other and the centromeres and telomeres located at opposite poles of the nuclei [89-91]. The presence of this organization is also known to vary greatly among tissues or developmental stages of an organism [90]. Then, one can hypothesize that this configuration might play a role in gene regulation through the partial decondensation of given chromosomal regions in specific tissues and at specific developmental stages, leading to the observed spatial partitioning of genes displaying similar expression profiles. This hypothesis is well supported by our previous results [33]. Indeed, a similar recombination- and expression breadth-based partitioning was found in barley in which the Rabl configuration is also observed, but not in maize which displays neither entirely Rabl nor entirely random chromosome organization [89,90].

## Conclusions

By combining the first reference sequence of a wheat chromosome with deep transcriptome sequencing data covering the whole plant development, we constructed a high density transcription map of the wheat chromosome 3B, comprising more than 8,800 transcriptionally active regions distributed throughout the entire chromosome. By studying the relationships between genome and gene structure and expression, we unraveled two interconnected mechanisms. The first one is a universal mechanism that relates to the ‘selection for economy’ and ‘genome design’ models and links gene structure and function, regardless of the gene position. The second one is an evolutionary force that links gene structure and function to gene position, leading to a strong partitioning of the wheat chromosome 3B. Since this partitioning is also observed in barley but not in other grasses, one can hypothesize that it has evolved with genome organization and is related to Triticeae-specific adaptation.

## Material and methods

### Sample preparation and sequencing

Total RNAs were extracted in duplicates from five organs (root, leaf, stem, spike, and grain) at three developmental stages each from hexaploid wheat *cv.* Chinese Spring [28] (Additional file 1: Table S3). RNA quality was assessed using an RNA nano Chip on the Agilent 2100 Bioanalyzer (Agilent Technologies, Santa Clara, CA, USA) and the RNA integrity number (RIN) was calculated for each sample. Only sample with a RIN greater than 7 were used for the library construction.

The 30 strand-non-specific RNA-seq libraries (representing the 15 conditions in duplicates) were constructed from 4 μg of total RNA using the Illumina TruSeq™ RNA sample preparation Kit (Illumina, San Diego, CA, USA) according to the manufacturer’s protocol, with a library insert size of 300 bp (fragmentation time of 12 min). Library profiles were evaluated using an Agilent 2100 Bioanalyzer. Illumina indexes were used to pool two samples per lane. Libraries were sequenced on an Illumina HiSeq2000 with 2 × 100-bp paired-end reads.

For strand-specific RNA-seq libraries, 12 μg of total RNAs from the same organ were pooled (4 μg per developmental stage) and polyA+ enriched RNAs were purified using the Ambion MicropolyA Purist Kit (Life Technologies, Carlsbad, CA, USA). Fifty nanograms of purified poly A+ RNAs were used to construct the oriented RNA-seq libraries with the ScriptSeq v2 RNA-seq Library Preparation kit (Epicentre, Madison, WI, USA) following the manufacturer’s instructions. After cDNA synthesis, 15 cycles PCR were performed to amplify the fragments. Libraries were purified by Ampure beads (Beckmann Coulter, Indianapolis, IN, USA) and then quantified using a Qubit Fluorometer (Life Technologies). Library profiles were evaluated using an Agilent 2100 Bioanalyzer. Each library was sequenced using 101 base-length read chemistry on one lane of a single-end (SE) flow cell on the Illumina HiSeq2000. Read quality was checked with the FastQC v0.10.0 software [92]. RNA-Seq data have been deposited under accession number ERP004714.

### Read alignment and expression analysis

Illumina reads were mapped on the chromosome 3B scaffolds using Tophat2 v2.0.8 [93,94] and bowtie2 [95] with the default parameters except: 0 mismatch, 0 splice-mismatch. PCR duplicates were removed with Samtools [96] rmdup option and an annotation-guided read alignment was performed with Cufflinks v2.1.1 [93,97] to reconstruct transcripts and estimate transcript abundance in units of fragments per kb of exon per million mapped reads (FPKM) [98]. Regions with FPKM values higher than zero were considered as expressed. TriAnnot-predicted regions were distinguished from unannotated regions (novel transcribed regions, NTRs) using the -g option. NTRs were reconstructed and ORFs were detected using transcripts_to_best_scoring_ORFs.pl (Trinity) [99] and blasted against the Magnoliophyta database (BLASTX, e-value 10e-5). Based on the FPKM scale defined in by Hansey and collaborators [79] expressed genes can be divided in three classes: genes with a FPKM value below 5 are low expressed, genes with a FPKM value greater or equal to 5 and less than or equal to 200 are medium expressed, and genes with a FPKM value greater than 200 are high expressed (semi-quantitative organization).

Sequences and annotations of the reference pseudomolecule and unassigned scaffolds have been deposited in ENA (project PRJEB4376) under accession numbers HG670306 and CBUC010000001 to CBUC010001450, respectively.

### Segmentation/change-point analysis

Segmentation analyses were performed using the R package changepoint v1.0.6 [100] with Binary Segmentation method and BIC penalty on the mean change. The different features that were subjected to this analysis were: recombination rate, transposable element density, predicted gene density, number of condition in which a gene is expressed. All these features were calculated in sliding windows of 10 Mb with a step of 1 Mb.

### Statistical analysis

All statistical analyses were performed with the R software [101]. Shapiro-Wilk test was used to test for normality of distribution. Correlation analyses were performed with Spearman rank correlation method. Outlier detection was performed using the formula: (Quantile 3 - Quantile 1) × 3 / Quantile 3, based on FPKM value and transcripts length of each gene. Genes were classified according to their average expression level and divided in 30 classes, with the same number of genes per class. R package ggplot2 was used to draw plot. Average comparison was performed using Welsh t.test to test for statistical significance between the five regions.

### Hierarchical clustering

Hierarchical clustering was performed using the Hierarchical Clustering Explorer 3.5 software [102] with the complete linkage method and the Pearson correlation coefficient. The minimal similarity to establish the clusters was set to 0.641 which is the Pearson correlation significant at the *P* value threshold of 0.01.

### Gene ontology enrichment analysis

GOBU (Gene Ontology Browsing Utility [103]) was used for enrichment calculations. The full set of 3B gene products annotated on the pseudomolecule [33] was used as the reference comparison set for the enrichment analysis of non-expressed genes. *P* values were calculated under GOBU with the Multiview Plugin and Fisher’s exact test.

## Additional files

Additional file 1: Table S1. GO term enrichment analysis of non-expressed genes. **Table S2.** Distribution of the 5,185 expressed protein coding genes according to their expression cluster and their chromosomal region. **Table S3.** Wheat RNA samples used for RNA-seq experiments. Additional file 2: Figure S1. Alternative transcript length as a function of expression level category. **Figure S2.** Expression breadth-based segmentation analysis of homoeologous chromosomes 3A and 3D. **Figure S3.** Relationships between gene expression and gene structural and functional features in the R1 / R3 regions. **Figure S4.** Relationships between gene expression and gene structural and functional features in the R2a / R2b regions.

## Abbreviations

A3SS: Alternative 3′ splice site
A5SS: Alternative 5′ splice site
AS: Alternative splicing
BLAST: Basic Local Alignment Search Tool
bp: Base pairs
*cis*-NAT: *cis*-natural antisense transcript
DNA: Deoxyribonucleic acid
FPKM: Fragments per kb of exon per million mapped reads
IR: Intron retention
kb: Kilobase pairs
lincRNA: Long intergenic non-coding ribonucleic acid
Mb: Megabase pairs
miRNA: Micro ribonucleic acid
mRNA: Messenger ribonucleic acid
MXE: Mutually exclusive exon
NAT: Natural antisense transcript
ncRNA: Noncoding ribonucleic acid
nt: Nucleotide
NTR: Novel transcribed region
ORF: Open reading frame
RNA: Ribonucleic acid
RNA-seq: Sequencing of RNA
TAR: Transcriptionally active region
TE: Transposable element
