# Supplementary material for: Deep transcriptome sequencing provides new insights into the structural and functional organization of the wheat genome
Source: Genome Biol. 2015 Feb 10;16(1):29. doi: 10.1186/s13059-015-0601-9 (PMC4355351; doi:10.1186/s13059-015-0601-9)
Supplement: Additional file 2: Figure S1. — Alternative transcript length as a function of expression level category. Figure S2. Expression breadth-based segmentation analysis of homoeologous chromosomes 3A and 3D. Figure S3. Relationships between gene expression and gene structural and functional features in the R1 / R3 regions. Figure S4. Relationships between gene expression and gene structural and functional features in the R2a / R2b regions. [file 13059_2015_601_MOESM2_ESM.docx]

**
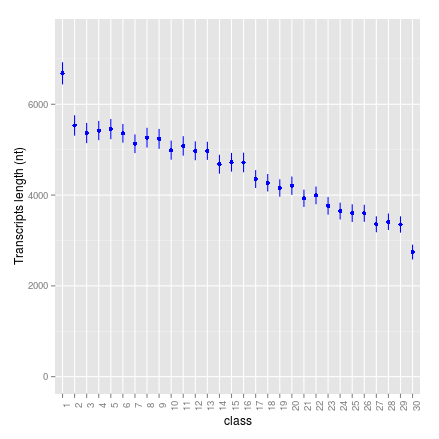
**

**Figure S1.** Alternative transcript length as a function of expression level category.

Expression levels are binned into 30 categories. Each dot is the mean value for genes in the given expression category, and the error bar indicates the standard deviation of the mean.


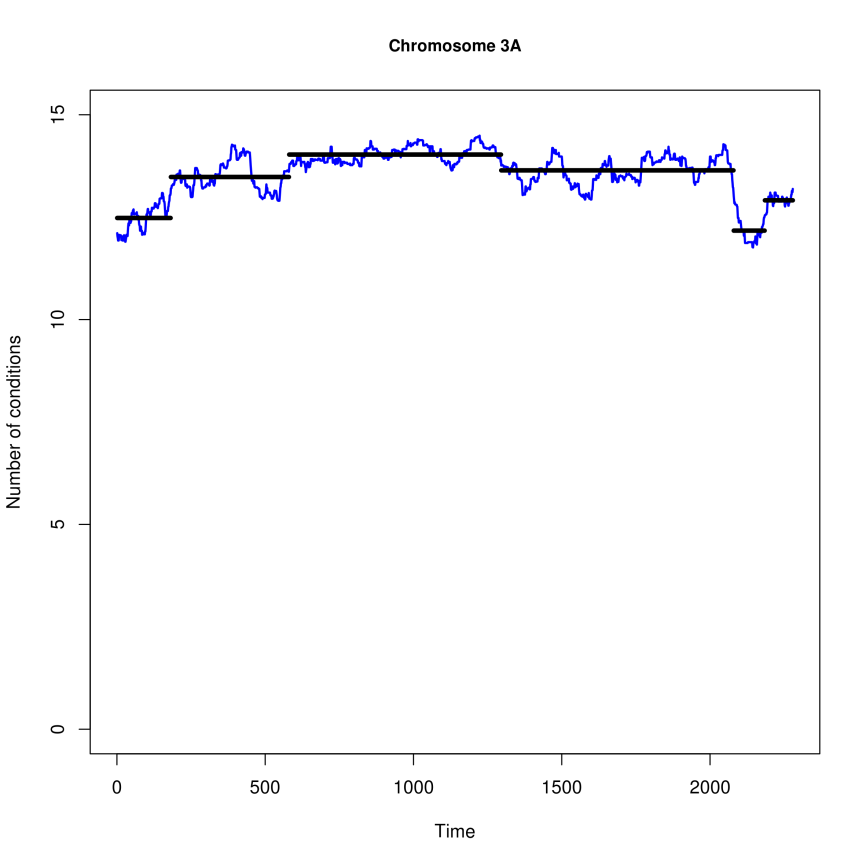

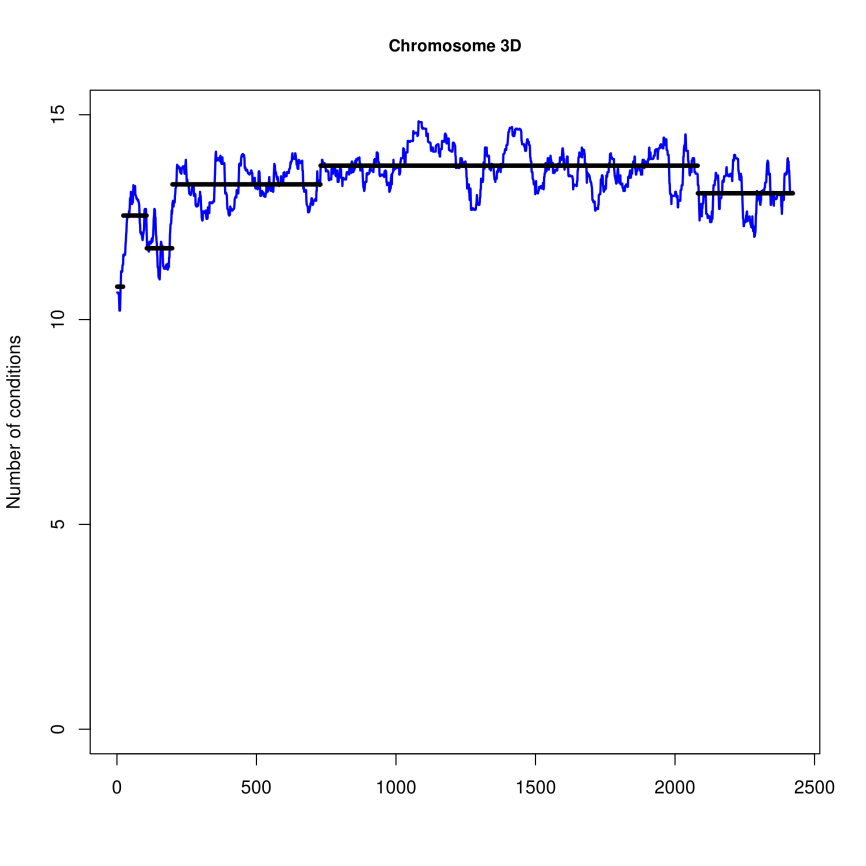


(B)

(A)

**Figure S2.** Expression breadth-based segmentation analysis of homoeologous chromosomes 3A and 3D.

Expression breadth is calculated as the average number of conditions in a sliding window of 50 genes. (A) chromosome 3A; (B) chromosome 3D.


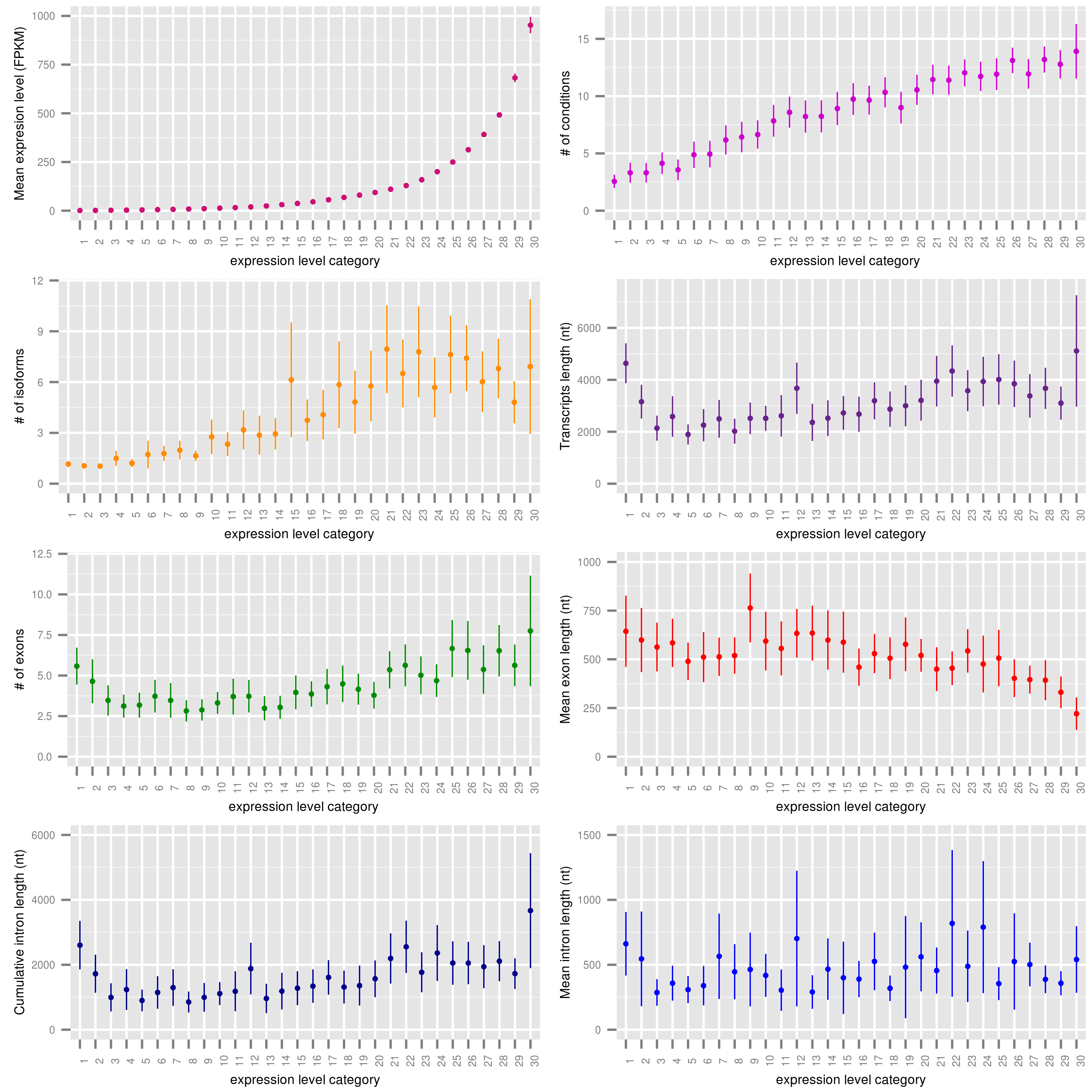


**Figure S3.** Relationships between gene expression and gene structural and functional features in the R1 / R3 regions.

Expression levels are binned into 30 categories. Each dot is the mean value for genes in the given expression category, and the error bar indicates the standard deviation of the mean.


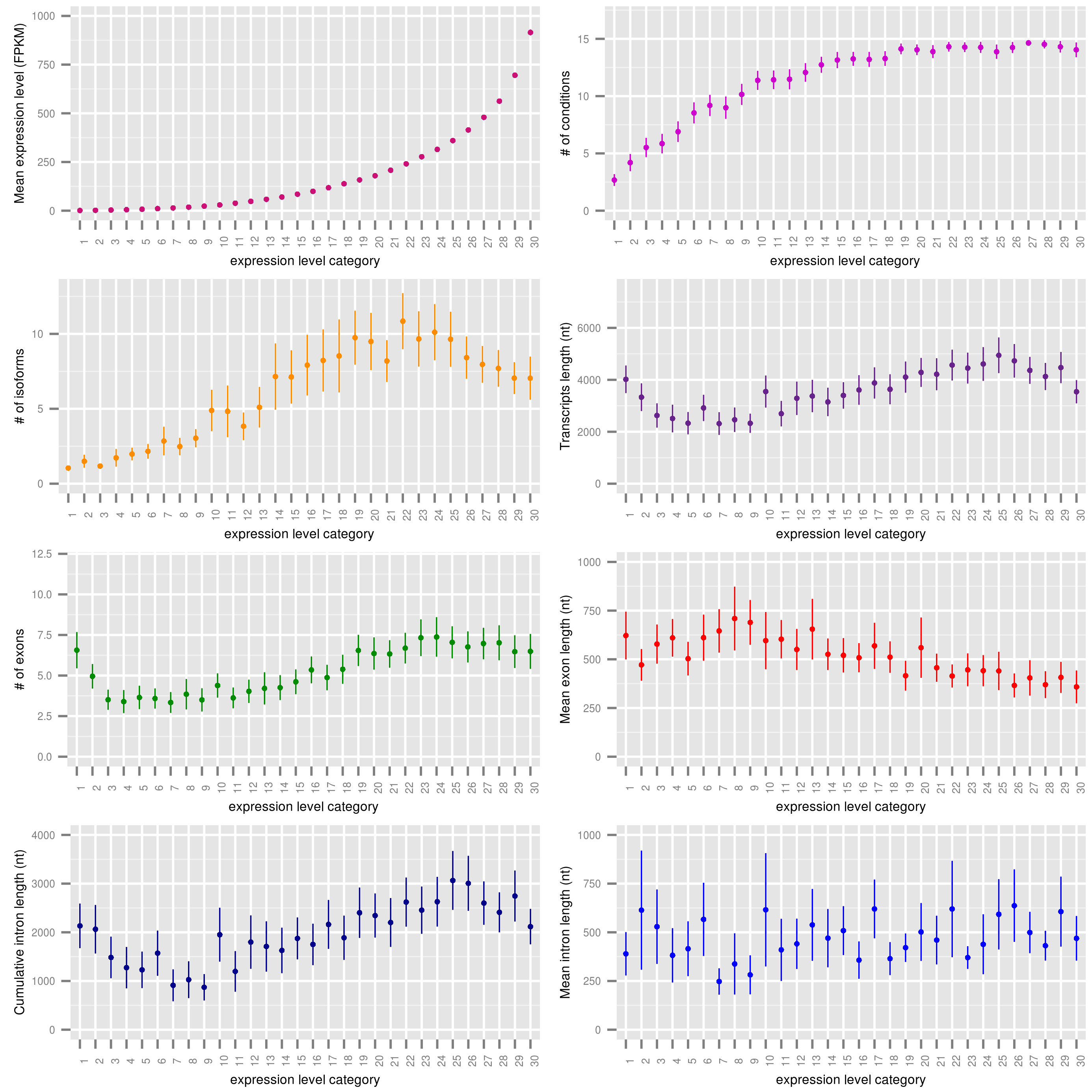


**Figure S4.** Relationships between gene expression and gene structural and functional features in the R2a / R2b regions.

Expression levels are binned into 30 categories. Each dot is the mean value for genes in the given expression category, and the error bar indicates the standard deviation of the mean.
